# Inflammation and stem markers association to PIM1/PIM2 kinase-induced tumors in breast and uterus

**DOI:** 10.18632/oncotarget.19438

**Published:** 2017-07-22

**Authors:** Manuel-Pedro Jiménez-García, Antonio Lucena-Cacace, María-José Robles-Frías, Irene Ferrer, Maja Narlik-Grassow, Carmen Blanco-Aparicio, Amancio Carnero

**Affiliations:** ^1^ Instituto de Biomedicina de Sevilla, IBIS, Hospital Universitario Virgen del Rocío, Universidad de Sevilla, Consejo Superior de Investigaciones Científicas, Seville, Spain; ^2^ Experimental Therapeutics Programme, Spanish National Cancer Centre (CNIO), Madrid, Spain; ^3^ CIBER de Cáncer, Instituto de Salud Carlos III, Pabellón 11, Planta 0, Madrid, Spain

**Keywords:** PIM kinases, conditional transgenic mice, oncogenesis, reproductive system tumors, mammary tumors

## Abstract

The PIM family of Ser/Thr kinase proteins has been implicated in tumorigenesis at different levels. PIM proteins are overexpressed in several tumor types and have been associated with chemoresistance. However, their role in hormone-dependent female tissues has not been explored, especially in the uterus, breast and ovary. We generated conditional transgenic mice with confined expression of human PIM1 or PIM2 genes in these tissues. We characterized the tumoral response to these genetic alterations corroborating their role as oncogenes since they induce hyperproliferation in all tissues and tumors in mammary gland and uterus. Furthermore, we observed a high degree of inflammatory infiltration in these tissues of transgenic mice accompanied by NFAT and mTOR activation and IL6 expression. Moreover, PIM1/2 were overexpressed in human breast, uterine and ovarian tumors, correlating with inflammatory features and stem cell markers. Our data suggest that PIM1/2 kinase overexpression provoke tissue alterations and a large IL6-dependent inflammatory response that may act synergistically during the process of tumorigenesis. The possible end-point is an increased percentage of cancer stem cells, which may be partly responsible for the therapy resistance found in tumors overexpressing PIM kinases.

## INTRODUCTION

The PIM (Provirus integration site for Moloney murine leukemia virus) family of serine/threonine kinases is composed of three different members, PIM1, PIM2 and PIM3, which are highly homologous. The expression of PIM genes is induced by a variety of cytokines, growth factors and mitogens [1]. The JAK/STAT transcription factors mediate the induction of transcription of these proteins that do not require posttranslational modifications for kinase activation [2, 3]. The stability and function of PIM kinases depend on protein levels achieved by a balance between translation and degradation [1, 3]. The PIM family of proteins has been implicated in the regulation of apoptosis, metabolism, cell cycle, homing and migration. PIM family members are oncogenes that can contribute to tumorigenesis at different levels [1, 3]. However, these proteins have been labeled as “weak oncogenes” given that their overexpression induces hyperproliferation but no tumors in many cases. Furthermore, these proteins have been shown to greatly enhance the capacity of other genes or chemical carcinogens to induce tumors [1, 3].

PIM kinases are overexpressed in hematological malignancies and solid tumors [4, 5], which has led to the postulation of these proteins as interesting targets for anticancer drug therapy [6–9]. *PIM-1* is frequently upregulated in hematopoietic malignancies, predicting poor prognosis [1, 2, 5, 10–12]. PIM-1 deregulation also occurs in bladder, gastric, colorectal and prostate carcinomas [13]. Recent studies have correlated PIM1 kinase with chemoresistance in prostate cancer cells [14, 15]. Therefore, PIM1 expression correlates with a poor therapeutic outcome [16]. PIM1 kinase is also associated with hypoxia-induced genetic instability in solid tumors, facilitating cell survival and resulting in tumors with a more aggressive phenotype [17]. Increased PIM1 expression could partly explain the strong resistance of these cancers to chemotherapy [18]. Increased PIM2 kinase levels have been detected in acute myeloid leukemia patients and B-cell derived malignancies [19, 20]. Furthermore, increased expression of PIM2 is also associated with aggressive clinical courses in ABC-DLBCL patients [21, 22]. PIM2 levels are also elevated in prostate cancers and are correlated with high proliferation and decreased apoptosis [23]. Aberrant expression of the PIM3 kinase was observed in malignant tumors of the liver and the pancreas and in Ewing's sarcoma [9, 24]. PIM3 is highly expressed in human hepatocellular carcinoma and pancreatic cancer lesions [25]. Finally, in some cancers, such as germ cell-derived tumors, all three PIM members are overexpressed [5], indicating that all three kinases share some common physiological properties. However, in these cancer types, PIM1 and PIM2 can be overexpressed in the same tumor, which suggests that there is only a partial redundancy among them. However, little information is known about the role of PIM kinases in female hormone-dependent tumors despite the fact that PIM1 is an estrogen-dependent transcript [26].

*Pim-1* is expressed in murine mammary tissue during all developmental stages [26]. *Pim-1* expression *in situ* is consistent with the documented profile of progesterone activity in mouse mammary glands. However, in human breast cancer cells, *PIM-1* is regulated by estrogen signaling, and its expression level contributes to the growth of mammary carcinoma cells. It has been recently described that PIM1 regulates cell death and chemotherapy response in triple-negative breast cancer [27]. Furthermore, elevated *PIM-1* expression is associated with malignant tumors and a higher tumor grade [26]. Even less information has been reported for the role of Pim kinases in other tissues, such as ovary and uterus, and its role in these organ tumors.

To characterize the proto-oncogenic role of PIM1/PIM2 in female hormone-dependent tissues, we generated two conditional transgenic mice with confined expression of human PIM1 or PIM2 genes in hormone-dependent tissues. We fully characterized the tumoral response to these genetic alterations in both PIM1 and PIM2 models, corroborating their role as oncogenes by inducing tumors in these tissues. More specifically, we detected tumors in mammary glands, ovary and uterus of transgenic mice overexpressing PIM proteins. Furthermore, we translated our discovery to human breast, cervix and ovary tumors analyzing public transcriptomic databases to study the relevance of these *in vivo* discoveries.

## RESULTS

### Phenotype of PIM1- or PIM2-expressing transgenic mice

We generated mouse lines that express PIM1 or PIM2 transgenes specifically in the female hormone-dependent organs by crossing our PIM1 or PIM2 transgenic lines with the MMTV-CRE transgenic line (Supplementary Figure 1A). Transgene expression was assessed using RT-PCR to confirm the specificity (Supplementary Figure 1B, 1C). Both transgenic lines were expressed only in the mammary glands, reproductive system tissues (uterus and ovary) and brain (Supplementary Figure 1B). The levels of transgenic PIM1 or PIM2 mRNAs are approximately ten- to twelve-fold increased on average compared with endogenous mouse Pim mRNAs (Supplementary Figure 1D).

To investigate the proto-oncogenic role of human PIM1 and PIM2 proteins in female hormone-dependent tissues, we generated conditional transgenic murine cohorts of female mice that overexpressed either PIM1 or PIM2 transgenic proteins. We observed a significant decrease in the average lifespan of the PIM transgenic cohorts, PIM1 and PIM2, compared with sibling WT cohorts (Supplementary Figure 1E). At the time of death, macroscopic analysis showed an increase in sexual organ alterations. However, PIM1 transgenic mice also exhibited a clear percentage of females with macroscopic mammary tumors (Supplementary Figure 1F). An in-depth microscopic analysis performed at necropsy on the sex organs revealed a clear increase in tumors in the transgenic mice (Supplementary Figure 1G). Specifically, up to 50% of the PIM1 and 30% of the PIM2 transgenic mice carried a tumor, whereas the percentage of WT mice with tumors did not reach 20%. At 700 days as the end-point, both transgenic lines had a statistically significant increase in the percentage of total tumors compared with WT mice (Supplementary Figure 1G).

### Hyperplasia and tumors induced by high expression of PIM kinases in the mammary gland

**Figure 1:**
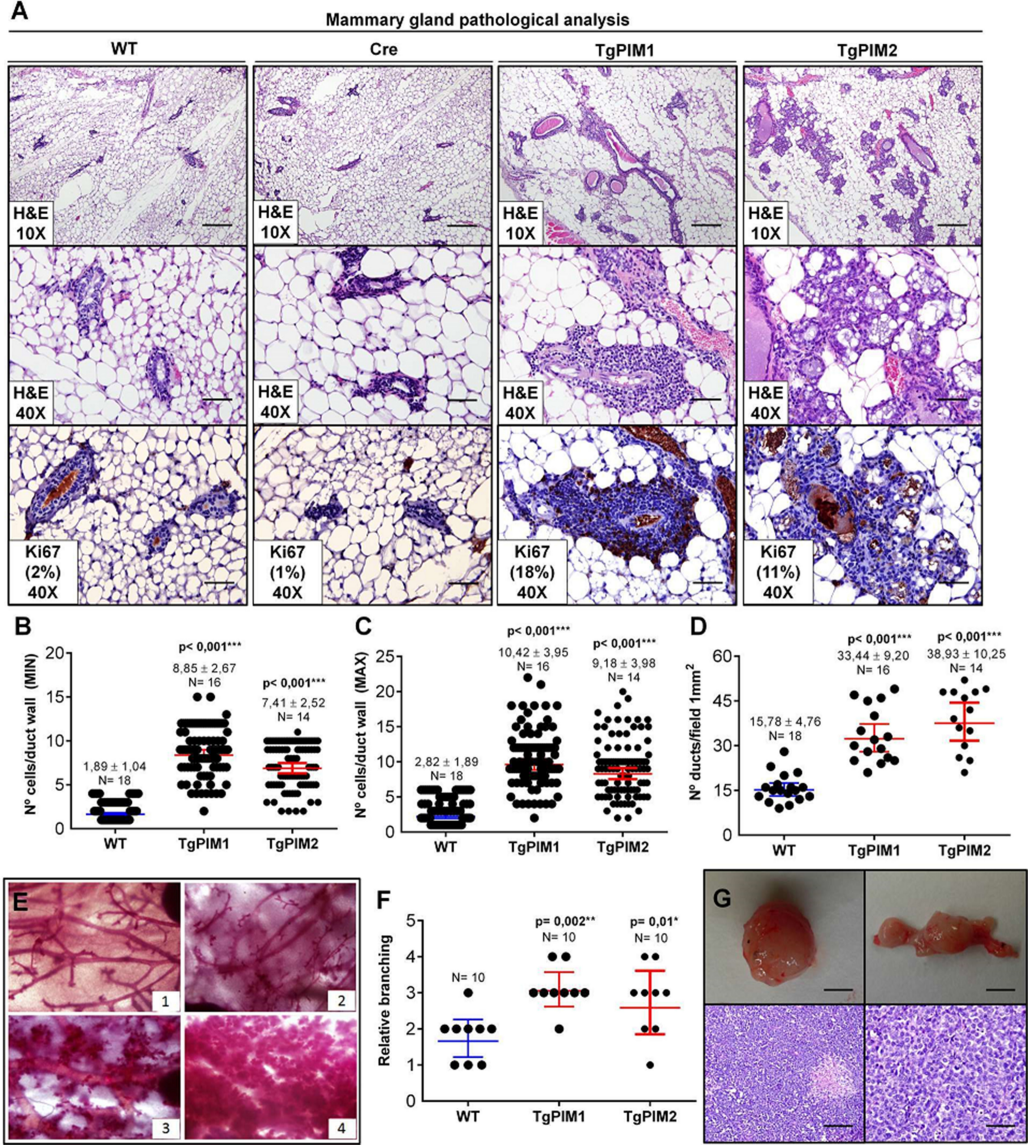
Pathological analysis of the mammary gland in TgPIM1/TgPIM2 transgenic models (**A**) Breast pathological analysis by Hematoxylin-Eosin staining (H&E) and Ki67 proliferation marker immunostaining of sections of the left hind leg mammary gland of both PIM models, WT and Cre mice. Representative images of neoplastic mammary lesions are presented at 10× (scale bars, 200 μm) and 40× (scale bars, 50 μm). (**B**–**C**) Average number of epithelial cell layers per mammary duct. To this end, layers of the opposite points of the perpendicular axis, MIN columns (B), and layers at the opposite points of the longest diameter, MAX columns (C), were considered, and the number of cell layers was counted. (**D**) Average number of mammary ducts per 1mm2 field of the two PIM models and WT. (**E**) Whole mammary gland mounts of mice were stained with carmine red alum to visualize the structure of the ducts and alveoli. Four different categories of phenotypes could be distinguished: (1) normal structure similar to the one of young virgin female mice; (2) greater than four-fold cystic dilation (of the ducts); (3) increased number of ramifications and strongly increased alveolar size; and (4) extreme alveolar hyperproliferation covering the space between the ducts to greater than 70%. (**F**) Mammary gland phenotypes of TgPIM1/TgPIM2 and WT mice. Points indicate the median of each mouse cohort classified according to (E). (**G**) Representative images of mammary gland tumors found in TgPIM1 mice. Upper images show a macroscopic view of tumors (scale bars, 1 cm). Lower images show H&E staining, left at 20× (scale bar, 100 μm) and right at 40× (scale bar, 50 μm). All images were captured by an Olympus BX-61 microscope and analyzed by Adobe Photoshop CS4. The *p*-value was obtained using the Wilcoxon signed rank test (*p < 0,05), (**p < 0,01) and (***p < 0,001).

In-depth analysis of the mammary glands revealed that transgenic PIM1 and PIM2 mice exhibited an increased percentage of cystic dilatation, alveolar growth and a clear increase in intraductal mammary neoplasia (MIN) (Figure 1A). Because these features could be the result of the abnormal proliferation of duct cells, we examined whether the increase in PIM proteins led to increased epithelial proliferation. We analyzed the mammary gland ducts of 9-week-old virgin transgenic and WT females by histology (Figure 1A–1D). First, we assessed the maximum and minimum number of epithelial cell layers of the mammary ducts for each mouse line. The average number of epithelial cell layers was increased in both PIM1 and PIM2 transgenic mice (Figure 1C). To avoid bias we also counted the number of epithelial cell layers at the thinnest point of each duct which was also significantly increased in PIM tg mice (Figure 1B). Next, we quantified the number of ducts per field (mm^2^) as a measure of increased proliferation. The number of ducts per field was also significantly increased in both PIM1 and PIM2 transgenic female mice (Figure 1D). Finally, we measured the relative branching found in the mammary grand of female mice as an indirect measure of mammary tumorigenic potential. We classified mammary branching arbitrarily in four stages (1–4), showing increased ducts per branch and alveolar proliferation (Figure 1E). We measured the branching of the female mice and classified them according to the branching stages established. Mammary glands from transgenic females exhibit significantly increased branching (Figure 1F). These results demonstrate that PIM transgenic mice display a number of pre-neoplastic abnormalities that are associated with increased cell proliferation and tumorigenic potential. This notion is clear given that 19% of PIM1 Tg female and a 7% of PIM2 Tg female harbored mammary tumors (Figure 1G). Surprisingly, the PIM2 transgenic cohort did not have tumors. We do not know whether this lower percentage of breast tumors in PIM2 Tg female is due to a lower oncogenic potential of PIM2 with respect to PIM1 or to the low number of mice analyzed (14 PIM2 vs. 16 PIM1). However, in all pre-neoplastic lesions, PIM2 seems to possess slightly lower proliferative capabilities than PIM1.

**Figure 2:**
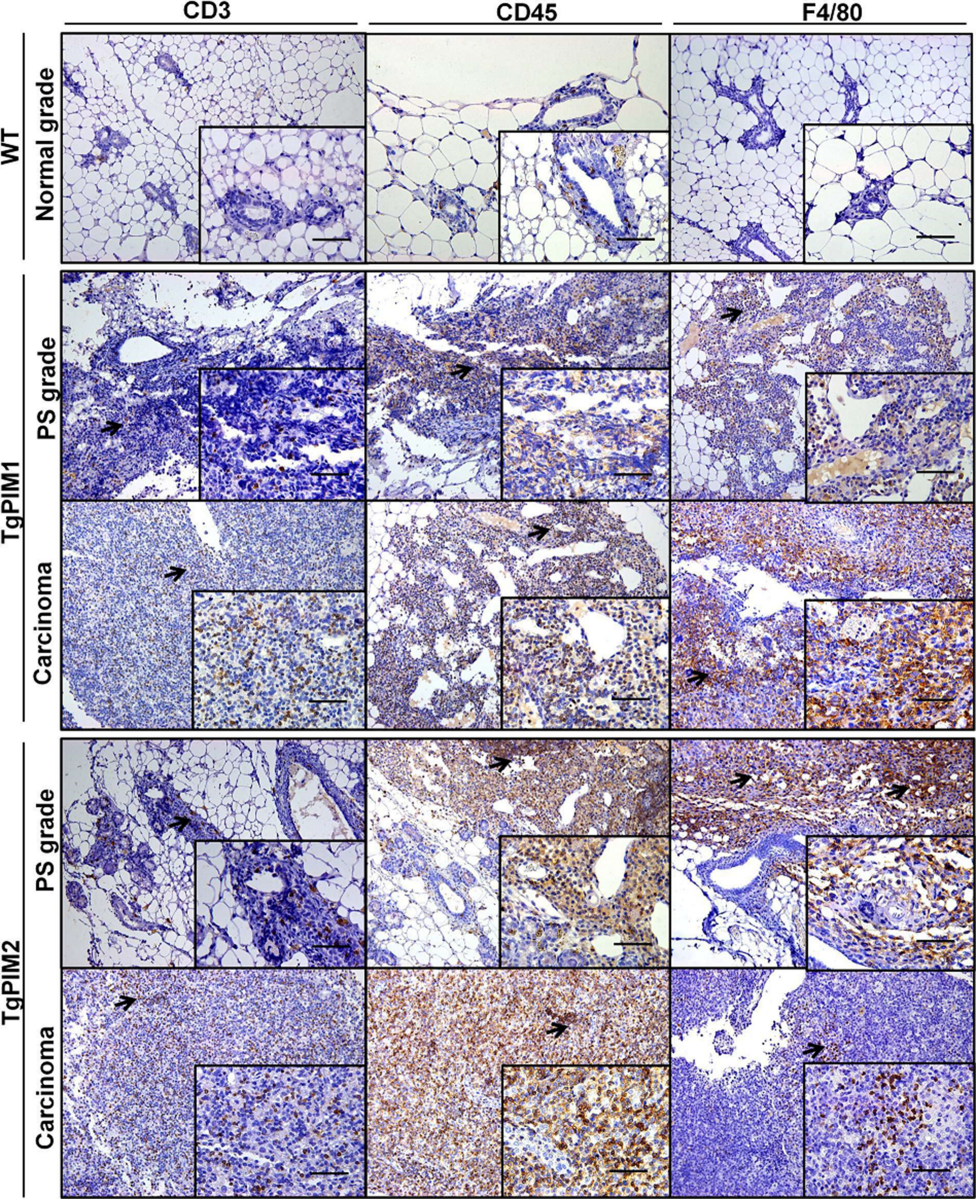
Immunohistological characterization of immune infiltration in the mammary glands from MMTV-Cre/PIM1 and MMTV-Cre/PIM2 transgenic models Pathological analysis of the mammary gland was performed by using immune mouse markers in order to determine whether the infiltration found in pathological analysis is lymphoid (CD3 and CD45) or macrophage (F4/80) origin. All markers are shown in WT (normal grade tissues), and both proliferative status grade (PS grade) and carcinoma for both TgPIM1/2 models. Scale bars indicate 50 μm (40×) in small-corned pictures and 100 μm (20×) in large pictures. The arrows highlight immune infiltration spots in each immune-marker. All of the images were captured using the Microscope Olympus BX-61.

Significantly, we observed a high degree of immune infiltration in the mammary gland of PIM transgenic mice, but not in WT mice. To confirm this point, we performed specific immunostaining of the mammary glands with common markers of the immune cells, CD3, CD45 and F4/80). We found clear and abundant immune infiltration in the mammary glands of the transgenic mice, either in hyperplasic or carcinoma tissues (Figure 2).

These data suggest that both PIM proteins may be involved in human breast tumor initiation. Our data also showed strong immune infiltration in these PIM-dependent tissues.

### Hyperplasia and tumors induced by high expression of PIM kinases in the uterus and ovary

**Figure 3:**
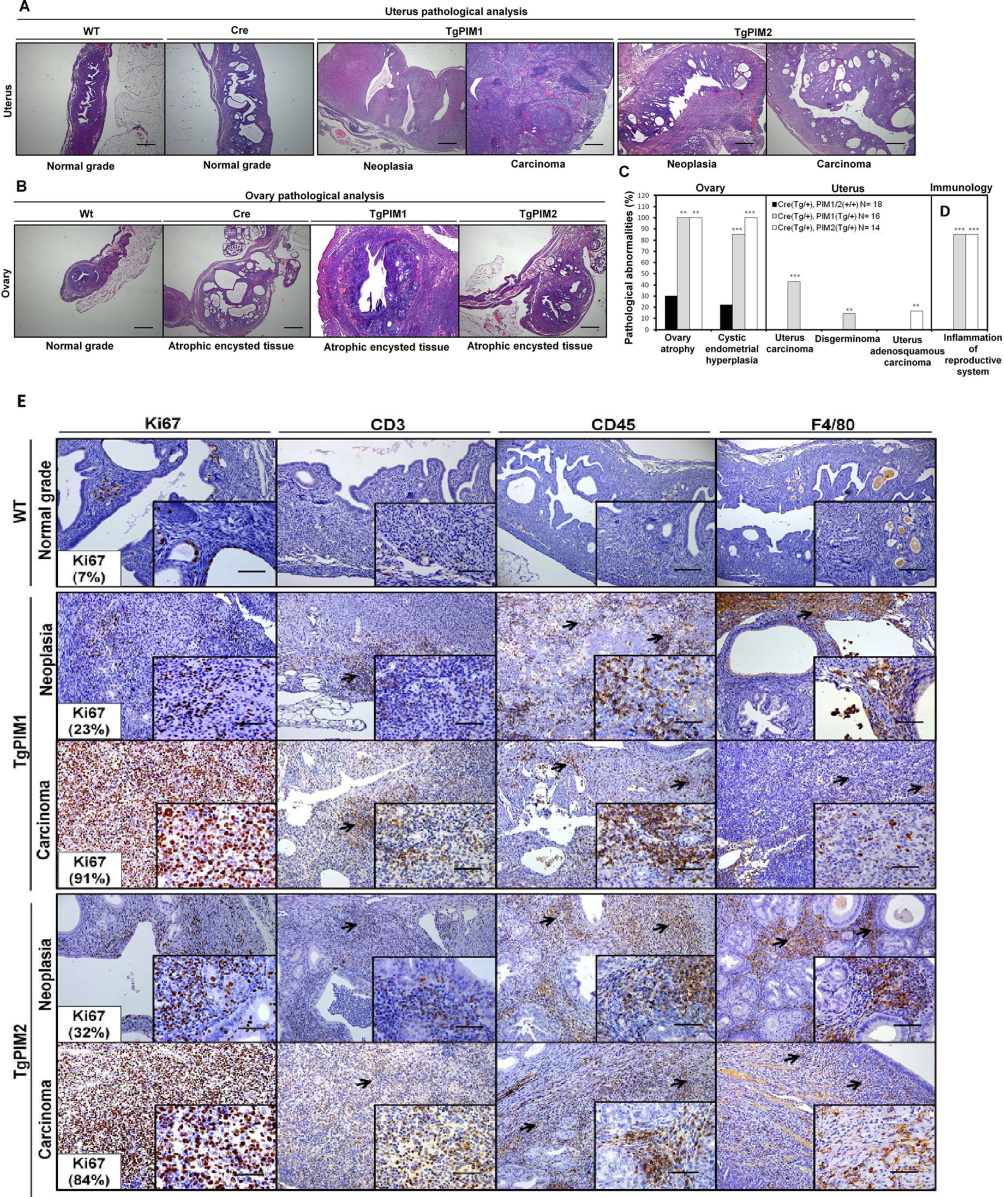
Pathological analysis of the uterus and ovary in TgPIM1/TgPIM2 transgenic models (**A**) Histopathological analysis of the uterus of WT and PIM1 and PIM2 transgenic females. Pictures depict representative observations in the different models. (**B**) Histopathological analysis of the ovary of WT, Cre and PIM1 and PIM2 transgenic females. Pictures illustrate representative observations in the different models. (**C**) Percentage of mice with pathological abnormalities observed. In each case, the statistics indicate the significance of the findings, compared to the WT females using Student's T test analysis, (*p < 0,05), (**p < 0,01) and (***p < 0,001). (**D**) Percentage of mice carrying inflammation in reproductive tissues. (**E**) Immunohistological characterization of immune infiltration in the uterus from MMTV-Cre/PIM1 and MMTV-Cre/PIM2 transgenic models. Pathological analysis of the uterus was performed by using both proliferation markers (Ki67) and immune mouse markers in order to determine whether the infiltration is lymphoid (CD3 and CD45) or macrophage (F4/80) origin. All markers are shown in WT, and both neoplasia and carcinoma stages for both transgenic models. The figure shows representative images of immune infiltration areas (marked with arrows in each marker). Scale bars indicate 50 μm (40×) in small-corned pictures and 100 μm (20×) in large pictures. All of the images were captured using the Microscope Olympus BX-61.

In-depth histopathological analysis of the uterus showed that most (90–100%) female transgenic mice had cystic endometrial hyperplasia (Figure 3A and 3C) either with PIM1 or PIM2. Approximately 50% of PIM1 transgenic females had malignant carcinoma, with 15% exhibiting disgerminoma. On the other hand, approximately 15% of PIM2 transgenic females harbored tumors, all of which were adenosquamous carcinoma (Figure 3A and C). On the other hand, 100% of transgenic females, of both PIM1 and PIM2 mice, exhibited ovary atrophy with clear hyperplasia (Figure 3B and C).

During the histopathological analysis, we observed a high degree of immune infiltration in the uterus and ovary in both transgenic cohorts but not in the WT mice (Figure 3C and 3D). This relationship between the expression of the PIM kinases and the mobilization of the immune system was strong because it was observed in approximately 100% of the transgenic mice. To confirm the immune infiltration in these tissues we performed specific IHC. Again we found significant signaling from CD3+, CD45+ and F4/80+ cells in both hyperplasia and carcinoma derived tissues from both transgenic lines (Figure 3F and 3G).

Our work is the first indicating that PIM1/PIM2 over-expression induces a neoplastic phenotype in the mammary gland and uterus, which indicates a possible role in the initiation of oncogenesis of PIM1 mainly but also PIM2 in these tissues. Furthermore, the presence of inflammation is likely an important second signal contributing to tumorigenesis.

### Expression of PIM1 and PIM2 in human tumors

**Figure 4:**
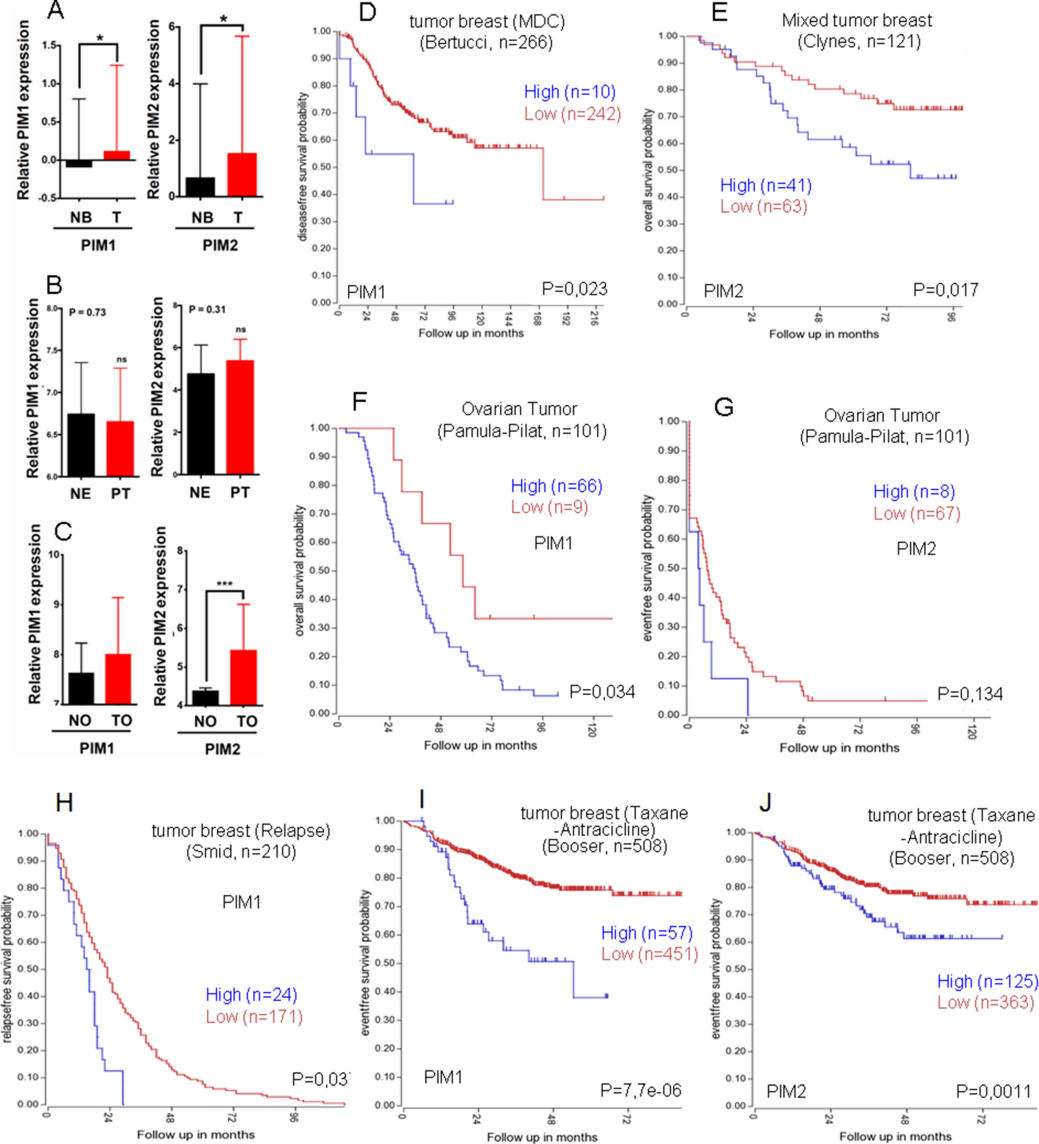
Relative PIM expression in human breast, endometrial and ovary tumors, Role in survival Relative PIM expression in human breast (**A**), endometrial (**B**) and ovary tumors (**C**). Patients were classified into groups based on the PIM1/2 gene expression level. A) Normal Breast (NB), Primary tumor (T); PIM1 *p* < 0,05 =*; PIM2 *p* <0,05 =*, by an unpaired *t*-test). B) Normal endometrium (NE), Primary tumor (PT); PIM1 *p* =0,73; PIM2 *p* = 0,31, by an unpaired *t*-test), ns = not statistically significant. C) Normal ovary (NO), Ovary tumor (TO); PIM1 *p* < 0,05; PIM2 *p* < 0,001 = ***, by an unpaired *t*-test), ns = not statistically significant. (**D**) Kaplan Meyer graph for disease free survival according to PIM1 levels in Breast cancer (public database used Bertucci, R2); (**E**) Kaplan Meyer graph for overall survival according to PIM2 levels in Breast cancer (public database used Clynes, R2); (**F**) Kaplan Meyer graph for overall survival according to PIM1 levels in ovarian cancer (public database used Pamula-Pilat, R2); (**G**) Kaplan Meyer graph for event free survival according to PIM2 levels in Ovarian cancer (public database used Pamula-Pilat, R2). (**H**–**J**) PIM overexpression in human breast tumors is indicative of worse prognosis in relapsed (H) or treatment resistant tumors (I-J). Kaplan Meyer graphs for relapse free survival from Public databases only from relapsed breast tumors (Smid, R2) according to PIM1 levels. (I, J). Kaplan Meyer graphs for event free survival from Public databases only from taxane and antracicline treated patients with breast tumors (Booser, R2) according to PIM1 (I) or to PIM2 (J) levels.

Analysis of human breast, endometrial or ovarian tumors from public databases revealed a variable subset of tumors overexpressing PIM1 or PIM2 (Figure 4A–4C). Interestingly, this subset with higher PIM1/2 expression has poor prognosis (Figure 4D–4G) that is also observed in relapse (Figure 4H) and resistance to therapy (Figure 4I, 4J), at least in breast tumors. We used several databases to shown that is not specific of one single database, but a rather common event, as has been shown for other solid tumors.

Thus, our data on transgenic mice complemented with human tumor analysis reinforces the role of PIM kinases as an antitumor target.

### PIM1 and PIM2 expression correlates with a proinflammatory phenotype in hormone-dependent female human tumors

To explore the possible PIM-dependent mechanism we analyzed human tumors. We selected public transcriptional databases of breast, endometrial and ovarian human tumors and analyzed genes that correlated with PIM kinases expression (R2: Genomics analysis and visualization platform (http://r2.amc.nl/), see Materials and Methods). We performed the analysis for each tumor type and then for genes common to all tumors. We found 665 genes which expression significantly correlated with the expression of PIM1 and PIM2 in human breast tumors (Pearson r > 0,3), 296 that significantly correlated to PIMs in endometrial tumors (Pearson r > 0,3) and 6661 in ovarian tumors (Pearson r > 0,3) (Supplementary Figure 2). Comparison of the genes that are common among these tumor types showed 80 genes common for al three tumors (Supplementary Figure 2, Supplementary Table 1). When we performed GO enrichment for Processes of the genes that correlated to PIMs expression we found a strong correlation to immune response (Supplementary Table 2).

**Figure 5:**
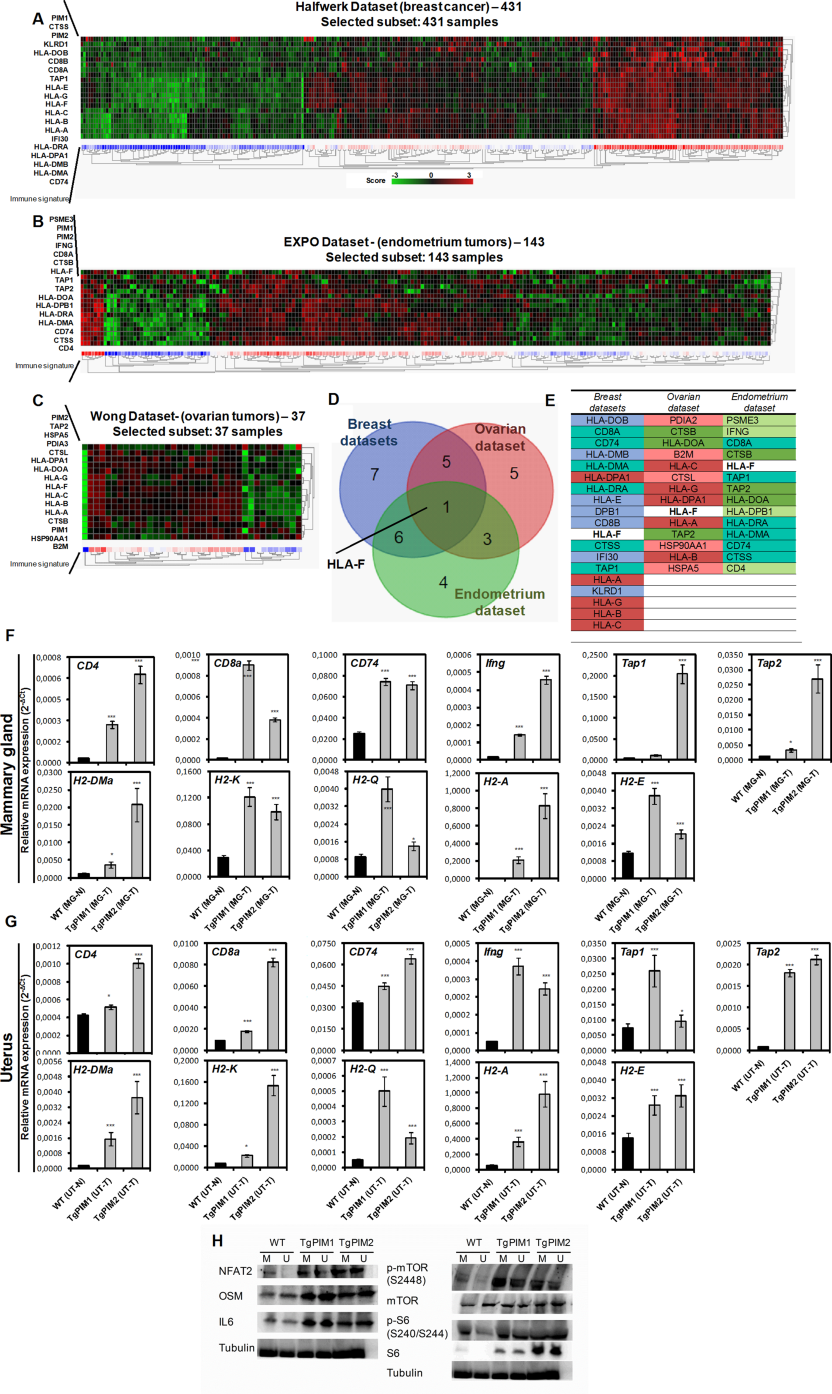
PIM overexpression correlates with an immune system-dependent genes signature in human breast, endometrium and ovary tumors (**A**) The figure shows a heat map comparing immunology-related genes with PIM1 and PIM2 (See Supplementary Table 3) in breast tumors (Halfwerk dataset, R2). Heat map shows a clear correlation of PIM1 and PIM2 with the expression of antigen presentation genes. (**B**) The figure shows a heat map comparing immunology-related genes with PIM1 and PIM2 (See Supplementary Table 4) in endometrial tumors (EXPO dataset, R2). Heat map shows a clear correlation of PIM1 and PIM2 with the expression of antigen presentation genes. (**C**) The figure shows a heat map comparing immunology-related genes with PIM1 and PIM2 (See Supplementary Table 5) in ovarian tumors (Wong dataset, R2). Heat map shows a clear correlation of PIM1 and PIM2 with the expression of antigen presentation genes. (**D**) A Venn diagram showing the number of immune system-dependent genes that correlated with PIM1 and PIM2 overexpression in Breast, endometrial and ovary tumors. (**E**) A list of system-dependent genes in Breast, endometrial and ovary tumors that correlated with both PIM1 and PIM2 overexpression (common) and that was unique to each tumor type. (**F**, **G**) Pro-inflammatory microenvironment PIM-dependent characterization by qPCR. The figure shows a number of immune system-dependent genes that were correlated with PIM overexpression in breast, endometrium and ovarian datasets: CD4, CD8a, CD74, Ifng, Tap1 and Tap2; and mouse Major Histocompatibility Complex markers (H2 complex). We decided to focus on the ones which has a mouse ortholog: HLA-DMA (H2-DMa), HLA-DRA (H2-A), HLA-DPB1 (CD74), and also H2-K (classical MHC class I), H2-Q (non-classical MHC class I), H2-E (classical MHC class II). They were measured by qRT-PCR of mammary gland of WT mice with non-proliferative status (MG-N) and carcinoma grades (MG-T) in both transgenic models (F), and the same way in uterus (UT-N, normal; and UT-T, carcinoma) (G). (**H**) The overexpression levels of the human transgenes PIM1 or PIM2 in the models causes an increase in the expression of NFAT and NFAT effectors (OSM and IL6), as well as the phosphorylation of mTOR. The *p*-value was obtained using a one-tailed student's *t*-test. (*p < 0.05), (**p < 0.01), and (***p < 0.001).

In all our studies with PIM models *in vivo*, we found a strong immune infiltration of PIM-induced hyperplasias and tumors. Therefore, we set to explore the relationship between PIM proteins expression and the immune system specifically (KEGG). These analyses showed a clear list of genes associated to antigen presentation that significantly correlated with PIM1 and PIM2 expression (Figure 5A–5C, Supplementary Tables 3, 4, 5) in all tumor types. Heat map of these genes clearly showed the correlation in breast, endometrial and ovarian tumors (Figure 5D, 5E).

Therefore, together with previous results, these correlations suggest that PIM-induced breast tumors may induce antigen presentation in these tumor cells which could act as immune-attractant.

To fully confirm these conclusions, we performed Q-RT-PCR in PIM tg mice to test whether the inflammatory molecules were already expressed in protumor lesions (Figure 5F and 5G and Supplementary Figure 3). We found clearly expressed HLA antigens in protumoral breast, ovary and uterus lesions in both transgenic mice, PIM1 and PIM2, (Figure 5F, 5G). Furthermore, other inflammatory antigens also present in human tumors, such as CD4, CD8a, CD74, IFNg, Tap1 or Tap2, also were activated in mice transgenic lesions (Figure 5F, 5G). Interestingly, most of these molecules were more activated in PIM1 than in PIM2 transgene, correlating with the relative oncogenic capability found in these mice.

Finally, to explore the mechanistic bases we measured the activation of the proteins NFAT and mTOR that might induce the expression of IL6, a well known chemo attractant of the immune system. NFAT2 belongs to a family of transcription factors that modulate the inflammatory response [28] as well as mTOR, while IL-6 has been connected to chronic inflammation, being produced at the site of inflammatory response [29]. In all the tumor tissues overexpressing PIM1/2, we detected a clear increment in the expression of IL-6, while NFAT2 was clearly increased and we observed an increase in two NFAT2 targets, cMYC and OSM. Furthermore, mTOR was phosphorylated and activated in tissues expressing PIM1/2 transgenes (Figure 5H), showing that PIM kinases have a real role as inflammatory inducers.

### PIM1 and PIM2 expression correlates with stem cell markers in hormone-dependent female human tumors

It is known that inflammation may trigger dedifferentiation of tumor cells towards a more stem-like phenotype. Moreover, PIM kinases are strongly associated with chemoresistance, and it is possible that factors related to self-renewal and pluripotency such as stem-like genes, which are triggered by PIM kinases, can partially explain the observed chemoresistance. Therefore, we tested whether PIM1 and PIM2 expression is correlated with stem markers in human female breast, uterine and ovarian tumors.

**Figure 6:**
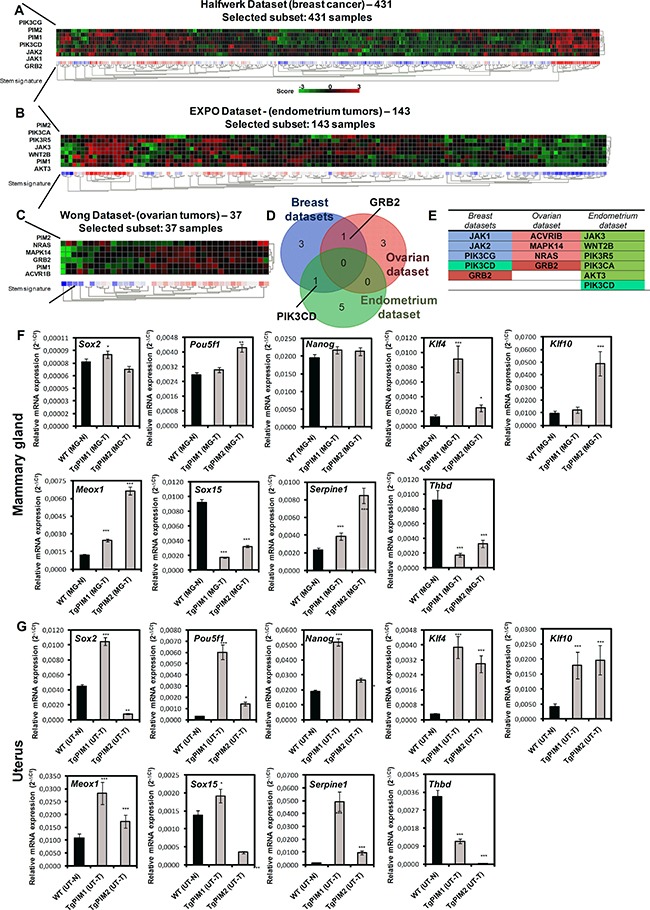
PIM overexpression correlates with stem-dependent genes signature in human breast, endometrium and ovary tumors (**A**) The figure shows a heat map comparing stem-related genes with PIM1 and PIM2 (See Supplementary Table 6) in breast tumors (Halfwerk dataset, R2). Heat map shows a clear correlation of PIM1 and PIM2 with the expression of antigen presentation genes. (**B**) The figure shows a heat map comparing stem-related genes with PIM1 and PIM2 (See Supplementary Table 7) in endometrial tumors (EXPO dataset, R2). Heat map shows a clear correlation of PIM1 and PIM2 with the expression of antigen presentation genes. (**C**) The figure shows a heat map comparing stem-related genes with PIM1 and PIM2 (See Supplementary Table 8) in ovarian tumors (Wong dataset, R2). Heat map shows a clear correlation of PIM1 and PIM2 with the expression of antigen presentation genes. (**D**) Venn diagram showing the number of stem-dependent genes that correlated with PIM1 and PIM2 overexpression in Breast, endometrial and ovary tumors. (**E**) List of system-dependent genes in Breast, endometrial and ovary tumors that correlated with both PIM1 and PIM2 overexpression (common) and that was unique to each tumor type. (**F**, **G**) Stem-related microenvironment PIM-dependent characterization by qPCR. The figure shows a number of stem-dependent genes that were correlated with PIM overexpression datasets (data not shown): Sox2, Pou5f1, Nanog, Klf4, Klf10, Meox1, Sox15, Serpine1 and Thbd. They were measured by qRT-PCR of mammary gland of WT mice with non-proliferative status (MG-N) and carcinoma grades (MG-T) in both transgenic models (F), and the same way in uterus (UT-N, normal; and UT-T, carcinoma) (G). The *p*-value was obtained using a one-tailed student's *t*-test. (*p < 0.05), (**p < 0.01), and (***p < 0.001).

To this end, we selected both PIM1 and PIM2 and explored the correlation between the expression of these kinases and the expression of stem-like genes activated by their expression (Figure 6, Supplementary Figure 4 and Supplementary Tables 6, 7, 8). We found a correlation between PIM1/2 expression and genes related to stem cell and pluripotency (Supplementary Tables 6, 7, 8). However, we observed more variability among tumors in these correlations according to the heat maps (Figure 6, Supplementary Figure 4) than in the immune response. Interestingly, in breast and endometrial tumors these genes are related to PI3K and JAK pathways, which suggest a combinatory therapy for the most resistant tumors related to PIM expression. In ovarian, however, this resistance seemed to be related to Ras pathway activation (Figure 6).

Finally, we performed Q-RT-PCR in PIM tg mice to test whether the stem cells related molecules were already expressed in tumor lesions (Figure 6F, 6G). We found clearly expressed molecules related to pluripotency, such as Sox2, Nanog, Klf4, or Serpine1, in tumoral breast, ovary and uterus lesions in both transgenic mice, PIM1 and PIM2, (Figure 6F, 6G). Therefore, our data confirmed that PIM1/2 kinase overexpression is a common feature of female hormone-dependent organs, which provoke tissue alterations and a large inflammatory response that may act synergistically during the process of tumorigenesis. There is also a correlation with markers of cancer stem cells, which may contribute to the therapy resistance found in tumors overexpressing PIM kinases.

## DISCUSSION

We generated conditional PIM1 and PIM2 transgenic mice that overexpress PIM1 or PIM2 in female hormone dependent tissues, namely mammary gland, uterus and ovary, and analyzed the contribution of these kinases to neoplastic initiation and progression. We found an increase in alterations in these organs, hyperplasia, and increased proliferation in the PIM1 and PIM2 transgenic mice. We also found a percentage of mammary gland and uterine tumors in PIM1 transgenic mice. The overexpression of PIM proteins also correlated with increased immune invasion in these tissues, perhaps by the expression of molecules immunoattractans by PIM overexpressing cells. We also found that PIM1 and PIM2 are overexpressed in a subset of human female breast, endometrial and ovarian tumors and that this overexpression correlated with clear inflammatory features and stem cell markers. Therefore, our data suggest that the overexpression of PIM1/2 kinases is a common feature of these female tumors with a large inflammatory response, which may act synergistically during the process of tumorigenesis. The possible end-point is an increased percentage of cancer stem cells, which may contribute to the therapy resistance found in tumors overexpressing PIM kinases.

At difference to other transgenic or knockout models, our model showed that increased expression of PIM1/2 alone, driven by the viral promoter MMTV, was sufficient to produce adenocarcinoma in breast and endometrium; moreover, PIM expression clearly contributed to the observed hyperplasia, which has been reported in other models. This finding is consistent with reports on cell lines that showed PIM overexpression alone was sufficient to enhance the *in vitro* and *in vivo* tumorigenic capabilities of tumor cells [30, 31]. In all cases, it seems that PIM2 possesses lower oncogenic capability than PIM1, at least in these specific female tissues analyzed in this work. Given the high degree of homology between both proteins and the redundant functions that both proteins seem to have in mice [32, 33], it is difficult to establish the nature of this difference. In line with this redundancy, the analysis in human tumors showed very similar pattern of transcriptional profile correlating with PIM1 or PIM2 genes (Figures 5 and 6). It is possible that minor differences in substrates account for the different oncogenic activity. On the other hand, it is possible that the poor oncogenic activity of PIM kinases is compensated with AKT kinase activity. It has been shown that AKT ser/thr kinase share many substrates with PIM kinases (6–9), and therefore may have similar input on tumorigenesis. This may explain the correlation with PI3K pathway found in human breast and endometrial tumors (Figure 6), a further activation in the PIM substrates may be necessary to develop full grown tumors, and AKT may supply this activity. Therefore, we may be looking to an additive network where full tumorigenic signaling may be supply as addition of kinase activity acting on similar substrates, PIM, AKT and MAPK. More research is needed to confirm this point.

PIM kinases have been implicated in chemoresistance in different types of cancer [34, 35], especially prostate [14, 15] and TNBC(27). We have shown that en human breast tumors, PIM levels are indicative of worse prognosis in Breast and endometrial tumors (Figure 5), and even among the tumors resistant to treatment with taxanes plus antracyclins, those with high levels of PIM kinases showed worse prognosis (Figure 5). This indicates that not only antiapoptotic networks are activated by PIM kinases.

In human breast, endometrial and ovarian cancer, a subset of tumors showed high levels of PIM1 and PIM2 correlating with inflammatory molecules. This data clearly correlates with our observations that in PIM transgenic mice, tumors and pretumoral lesions are accompanied by immune infiltration. Therefore, our data suggest that a large inflammatory response is a common feature of those tumors with overexpression of PIM1/2 kinases due to tissue organ alterations. This inflammatory response may act synergistically during the process of tumorigenesis. It is possible that the expression of the PIM proteins acts as an immune chemoattractant, and this immune presence then collaborates with neoplastic alterations.

In this regard, enhanced inflammation surrounding target tissues could be a tumor promotion mechanism led by PIM deregulation of cellular JAK/STAT signaling because STAT proteins have been intimately tied to controlling the development of hematopoietic cells that regulate inflammation, and mediate the responses of target cells to inflammatory cytokines. PIM has been implicated in inflammation in several *in vitro* and *in vivo* models [36, 37]. PIM1 and PIM2 have also been shown to belong to an endogenous pathway that regulates T-cell growth and survival [38, 39]. PIM1 has been shown to regulate human Th1 cell differentiation [40] and play a role in immune cell activation and proliferation [41]. In addition, PIM1 appears to contribute to NFκB activation upon TNF-α activation [42] through a feedback loop, while PIM1-inhibitors prevent NFκB activation and decrease iNOS production in macrophages and decrease levels of TNFα.

Mechanistically, high PIM levels increase the recruitment of tumor/inflammation associated macrophages, MDSCs, mast cells, and neutrophils to the target tissue, by increasing IL-6 locally as well as other cytokines (such as OSM) and probably other chemokines (such as CCL2 and CXCL8). It is possible that the increase in these cytokines in the extracellular media surrounding tumor cells might promote tumorigenesis by activating the NFkB and/or STAT3 pathways [43].

The correlation between human breast, endometrial and ovary tumors with a stem cell signature is also very interesting. Elevated PIM kinase levels promote the self-renewal of stem cells [44, 45] and may cooperate in cell reprogramming upon LIF stimulation [46]. PIM kinases might influence self-renewal and reprogramming through various pathways, such as directly phosphorylating BCRP/ABCG2, a putative stem cell marker, which also may be involved in multiple drug resistance [47]. PIM consistently phosphorylates and enhances OCT4 and MYC, which also contributes to the reprogramming of tumor cells [48, 49]. It is interesting to remark that the common pathways involved in this stem cell signature correlating to PIM expression are related to AKT and MAPK.

Alternatively, PIM may influence self-renewal and reprogramming through enhanced inflammation, which is observed in the tumor microenvironment. Monocytes and macrophages recruited to the tumor directly regulate cancer stem cells (CSC) through inflammatory cytokines IL-1, IL-6, and IL-8, which drive CSC self-renewal in their niches. These cytokines activate the STAT3/NF-kB pathways in tumor and stromal cells generating positive feedback loops that contribute to CSC self-renewal [50]. Growth factors such as EGF also activate NFkB. Moreover, chemokine receptors and their ligands are frequently expressed in malignant cells [51]. Non-CSCs may revert to a stem cell-like state, even in the absence of DNA mutations, by inflammatory released molecules, which confirms that the microenvironment is able to regulate the stem-like properties of cells [52].

In summary, we fully characterized transgenic mouse models expressing high levels of PIM kinases in breast, endometrium and ovary, corroborating their role as oncogenes. PIM1/2 overexpression induced hyperproliferation and tumors in the female reproductive system. Furthermore, we found a high inflammatory response and stem markers associated with PIM expression. The analysis of human breast, endometrial and ovarian tumors showed that PIM overexpressing tumors are also associated with an increased antigen presentation and immune response and the presence of stem cell markers. We propose that PIM kinases induce a pro-inflammatory microenvironment that cooperates with PIM kinases in the tumorigenic process by either directly or indirectly modifying the CSC pool.

This relationship between PIM kinases and immune mobilization of the immune system is not unknown, as there are numerous references related to this issue. However, limited examples of *in vivo* murine models showing the relationship between PIM kinases and immune infiltration are limited. Additionally, enhanced inflammation surrounding target tissues could be a tumor initiation mechanism led by PIM deregulation of cellular JAK/STAT signaling given that STAT proteins are intimately tied to controlling the development of hematopoietic cells that regulate inflammation and mediate the responses of target cells to inflammatory cytokines.

## MATERIALS AND METHODS

All methods were performed in accordance with the relevant guidelines and regulations of the Institute for Biomedical Research of Seville (IBIS) and University Hospital Virgen del Rocio (HUVR).

### Maintenance of mouse colonies

All of the experiments performed using animals received expressed approval from the IBIS/HUVR Ethical Committee for the Care and Health of Animals. All of the animals were maintained in the IBIS animal facility according to the facility guidelines, which are based on the Real Decreto 1201/2005, and were sacrificed by CO_2_ inhalation either within a programmed procedure or as a humane endpoint when the animals showed significant signs of illness. Efforts were made to minimize suffering wherever possible.

Primers designed specifically for human PIM1 and PIM2 kinases that do not amplify mouse Pim genes were used for all PCR experiments and subsequent genotyping of mice. PIM1 Fw: 5′: CGAGATCGCCATATTTGGTGTCCCCGAG; Rv: 5′: CCAGCTTGGTGGCGTGCAGGTCGTTGCA; PIM2 Fw: 5′: GGCAGCCAGCATATGGG; Rv: 5′: TAATC CGCCGGTGCCTGG. For mouse genes: Pim1 Fw: 5’: CAAGGACGAAAACATCCTTATC; Rv: 5′: GATG GGACCCGAGTGTATAGCC; Pim2 Fw: 5′: GGCAG CCAGCATATGGGC; Rv: 5′: TAATCCGCCGGT GCCTGG.

### Necropsy

All of the tissues and tumor samples were immediately dissected after death, fixed in 10% buffered formalin for 24 h, dehydrated at different ethanol concentrations with xylol and embedded in paraffin at 65°C to obtain tissue blocks.

### Whole mounts

Mammary glands were harvested, placed on dry xylanized glass slides and fixed overnight in 1:3:6 parts glacial acetic acid:chloroform:100% ethanol. Tissues were rehydrated through successive incubations with 70% ethanol followed by distilled water and stained with carmine red alum overnight. Tissues were then dehydrated through incubations in graded ethanol followed by mixed xylenes and were mounted in Permount® (Fisher Scientific).

### Immunohistochemistry

Paraffin blocks were cutted into 2.5 μm sections, mounted and dried on glass slides. Sectioned tissues were deparaffinized in xylol, followed by dehydration in graded alcohol solutions and were stained with hematoxylin-eosin (H&E). Epitope antigen retrieval was performed in sodium citrate (pH 6.5). Endogenous peroxidase activity was blocked using DAKO blocking solution for 20 minutes at room temperature. Non specific protein binding was saturated using PBS+10% FBS, 1% BSA and 0.3% Triton X-100 for 1h at room temperature. The primary antibodies (Ki67, CD3, CD45 and F4/80) were incubated overnight at 4°C. A secondary antibody anti-goat (ab97100) for: CD3 (sc-1127); anti-rat (JI-112-035-003) for: CD45 (NB110-93609) and F4/80 (MCA497), and anti-rabbit (JI-111-035-003) for: Ki67 (Master diagnostica), was applied for 1 h at room temperature, and the immunocomplexes were revealed using substrate buffer and chromogen (Envision, Flex DAKO). The tissues were counterstained with hematoxylin (DAKO), rehydrated in a graded alcohol series, and mounted using coverslips.

Analysis of relative (RT-PCR) and quantitative (qRT-PCR) expression of PIM1/PIM2 mRNA transgenes and immune- and stem-dependent mRNAs

Total RNA was isolated using miRNeasy kit (Qiagen), and reverse transcribed. We used the following TaqMan Gene Expression Assays probes (ThermoFisher) after RT-PCR: PIM1 (Hs01065498_m1), PIM2 (Hs00179139_m1), Pim1 (Mm00435712_m1), Pim2 (Mm00454579_m1), Meox1 (Mm00440285_m1), Sox15 (Mm00839542_g1), Pou5f1 (Mm03053917_g1), Nanog (Mm02019550_s1), Sox2 (Mm03053810_s1), Klf4 (Mm00516104_m1), Klf10 (Mm00449812_m1), Serpine1 (Mm00435858_m1), Thbd (Mm00437014_s1), CD4 (Mm00442754_m1), CD8a (Mm01182107_g1), CD74 (Mm00658576_m1), Ifng (Mm01168134_m1), Tap1 (Mm00443188_m1), Tap2 (Mm01277033_m1). The main HLA in mouse (H2s): H2-DMa (Mm00439226_m1), H2-K (Mm01612247_mH), H2-Q (Mm00657093_g1), H2-A: (Mm00439211_m1), H2-E: (Mm00772352_m1), and endogenous housekeepings: GADPH (Hs03929097_g1) and Gadph (Mm99999915_g1). Real-time PCR was performed using an ABI 7900HT (ThermoFisher). The relative mRNA quantities were expressed as 2^-ΔCt^. Relative mRNA quantification and statistical analysis of qPCR data were conducted using RQ Manager 1.2.1 software (ThermoFisher).

### Western blot analysis

Western blot analyses were performed as previously described (Carnero and Beach 2004, Castro et al. 2008). Total protein extract was isolated from N_2_ pulverized tissues. The tissues were lysed by sonication in lysis buffer (50 mM Tris-HCl, pH 7.5; 1% NP-40; 1 mM Na_3_VO_4_; 150 mM NaCl, 20 mM Na_4_P_2_O_7_; 100 mM NaF; 1% Na-deoxycholate; 0.1% SDS; 1 mM EDTA; phosphatase inhibitor cocktail (Sigma) and protease inhibitor cocktail (Sigma)). The samples were quantified and 30 μg were separated on 4–15% SDS-PAGE gels, transferred to Nitrocellulose membranes (Amersham) and inmunostained. The following primary antibodies were used at 1:1000 dilution: anti-NFAT2 (#8032, CST), anti-OSM (AF-495, R&D), anti-IL6 (#12912, CST), anti-pmTOR(S2448) (#5536, CST), anti-mTOR (#2983, CST), and anti-α-tubulin (#9026, SIGMA) at 1:10.000 dilution. A secondary antibody horseradish peroxidase-labeled were used according to primary antibody host at 1:5.000 in every case and 1:10.000 in anti-α-tubulin: rabbit anti-mouse (ab97046, Abcam), goat anti-rabbit (ab97051, Abcam), rabbit anti-goat (ab97100, Abcam). The proteins were visualized by using ECL detection system (Amersham).

### Statistical analysis

All of the grouped data are presented as the mean ± standard error. The differences between the groups were assessed by a one- or two-tailed Student's *T*-test using GraphPad Prism Software. For survival analysis, Kaplan-Meier curves were generated using Prism Software, and a long-rank test was performed to determine significant differences between the groups. All of the experiments were repeated in at least duplicate with triplicate technical replicates for each condition. The data distribution was assumed to be normal, but this was not formally tested. The data obtained for retrospective analysis were collected and processed in appropriate experimental arms.

### Bioinformatic analysis

We have analyzed the available, public tumor datasets for PIM kinases expression in breast tumors (GSE6532, GSE3494, GSE1456, GSE7390, GSE5327), endometrium tumors (EXPO209, GSE2109) and ovarian tumors (GSE40595) available at Oncomine (Compendia Biosciences, www.oncomine.org) and R2: Genomics analysis and visualization platform (http://r2.amc.nl). High and low groups were defined as above and below the mean respectively. For analysis with high and low groups, high was defined as greater than one standard deviation above the mean, low is greater than one standard deviation below the mean. We also use independent datasets of breast, endometrium and ovarian cancers (Clynes dataset: GSE42568; Booser dataset: GSE25066; Smid dataset: GSE29271; and Bertucci dataset GSE21653) for all Kaplan-meier analysis. One-way ANOVA was performed to get the statistics. Heat-meaps are sorted by City-block distances.

## SUPPLEMENTARY FIGURES AND TABLES




