# Supplementary material for: Inflammation and stem markers association to PIM1/PIM2 kinase-induced tumors in breast and uterus
Source: Oncotarget. 2017 Jul 22;8(35):58872–86. doi: 10.18632/oncotarget.19438 (PMC5601700; doi:10.18632/oncotarget.19438)
Supplement: Supplementary file 1 [file oncotarget-08-58872-s001.pdf]

Inflammation and stem markers association to PIM1/PIM2 kinase-induced tumors in breast and uterus

SUPPLEMENTARY MATERIALS

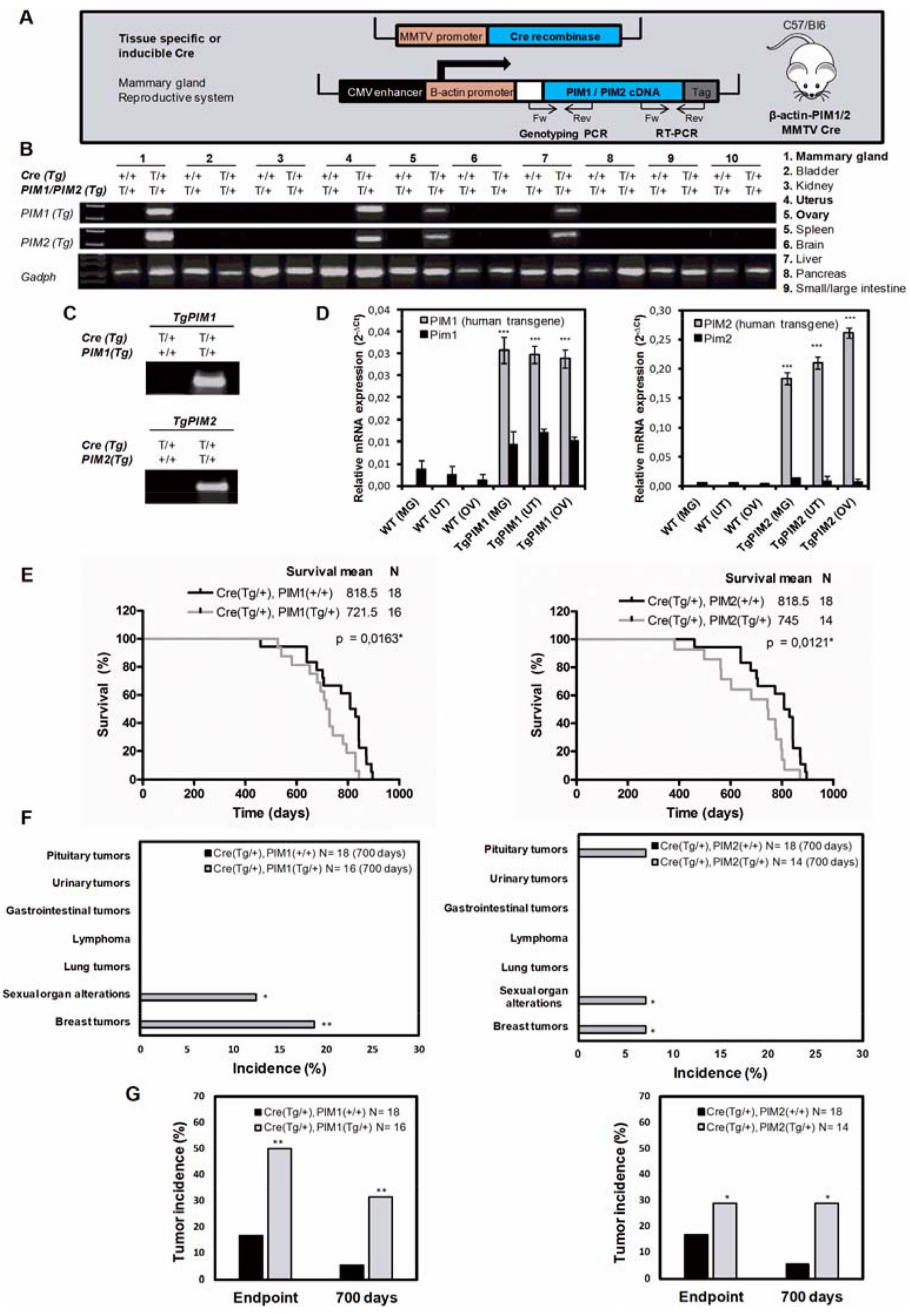

Supplementary Figure 1: Generation and validation of transgenic MMTV-Cre/PIM1 and MMTV-Cre/PIM2 conditional murine models. (**A**) Animals carrying respective PIM1/PIM2 transgenes silenced by a stop sequence flanked by LoxP sites were crossed with mice expressing Cre-recombinase regulated by the MMTV promoter. The result is two mouse lines that express either PIM1 or PIM2 human transgenes specifically in hormone-dependent tissues, where Cre expression excised the Lox/Stop/Lox cassette and allowed transgene expression. (**B**) Reverse transcriptase PCR was performed to validate tissue-specific relative expression levels of either PIM1 or PIM2 in TgPIM1/TgPIM2 models, indicating specific transgene expression in the mammary gland, female reproductive organs (uterus and ovary) and brain tissues. (**C**) DNA obtained from the digested tails of the mice was used to genotype the TgPIM1 or TgPIM2 models by PCR. (**D**) Levels of PIM1/PIM2 transgene relative to Pim1/Pim2 endogenous mRNAs were measured in mammary glands (MG), uterus (UT) and ovary (OV) of both TgPIM1/TgPIM2 and WT mice by qRT-PCR. (**E**) TgPIM1, TgPIM2 and WT model mouse survival as indicated by Kaplan-Meier curves. Both models exhibit a statistically significant reduction of survival rate compared with WT mice. (**F**) Tumor incidence in both female models at day 700. Data show the observed macroscopic pathologies in TgPIM1 and TgPIM2 mice compared with WT background, indicating a statistically significant tumor incidence increase in sexual organ alterations in both models. (**G**) Total tumor incidence in both models at day 700 and the clinical endpoint. This graph shows total percentage of tumors observed from a macroscopic view per model compared with WT, indicating a statistically significant increase in the percentage of total tumor types in the TgPIM1/TgPIM2 models. The p-value was obtained using a one-tailed Student's *t*-test in (A, B, C, D, F and G). The *p*-value was obtained using a Mantel-Cox test (also called Log rank Chi2-based test) in (E), (*p < 0,05), (**p < 0,01) and (***p < 0,001).

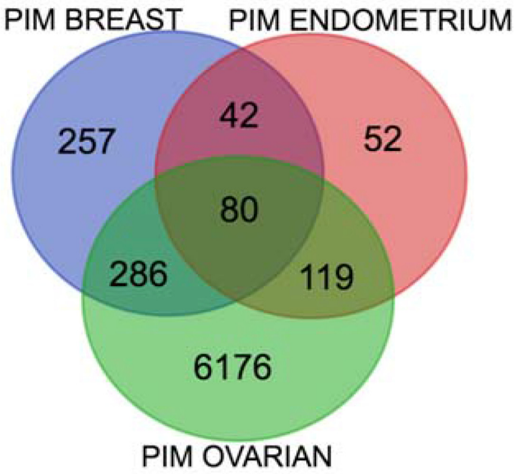

Supplementary Figure 2: Venn-diagram of genes that correlate with PIM1 and PIM2 in Breast, endometrial and ovarian tumors, and genes common to combination of tumor types.

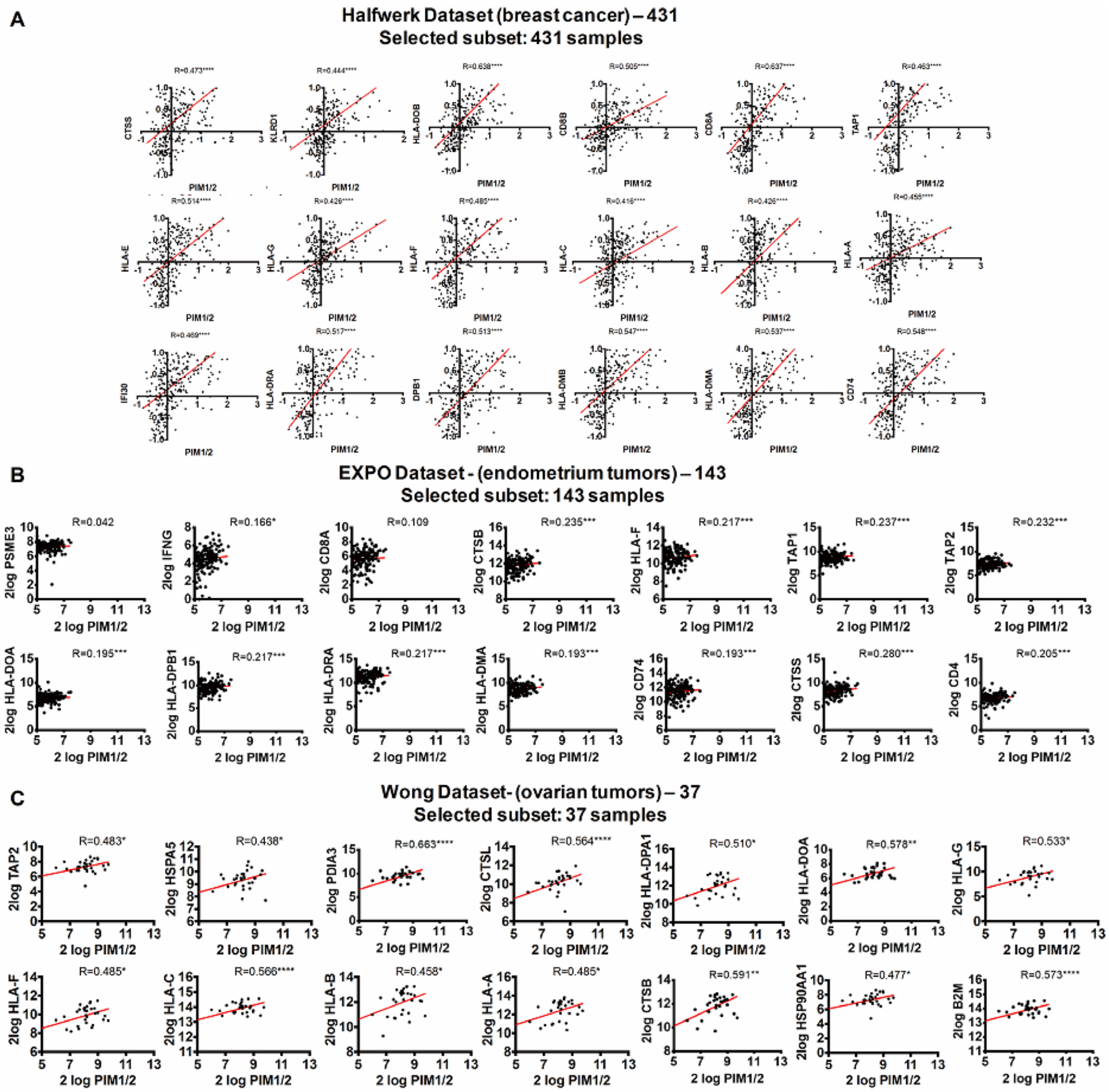

Supplementary Figure 3: One to one Pearson's correlation of the list of immune system-dependent genes signature depicted in the heatmaps displayed in Figure 5 of human breast, endometrium and ovary tumors databases analysed.

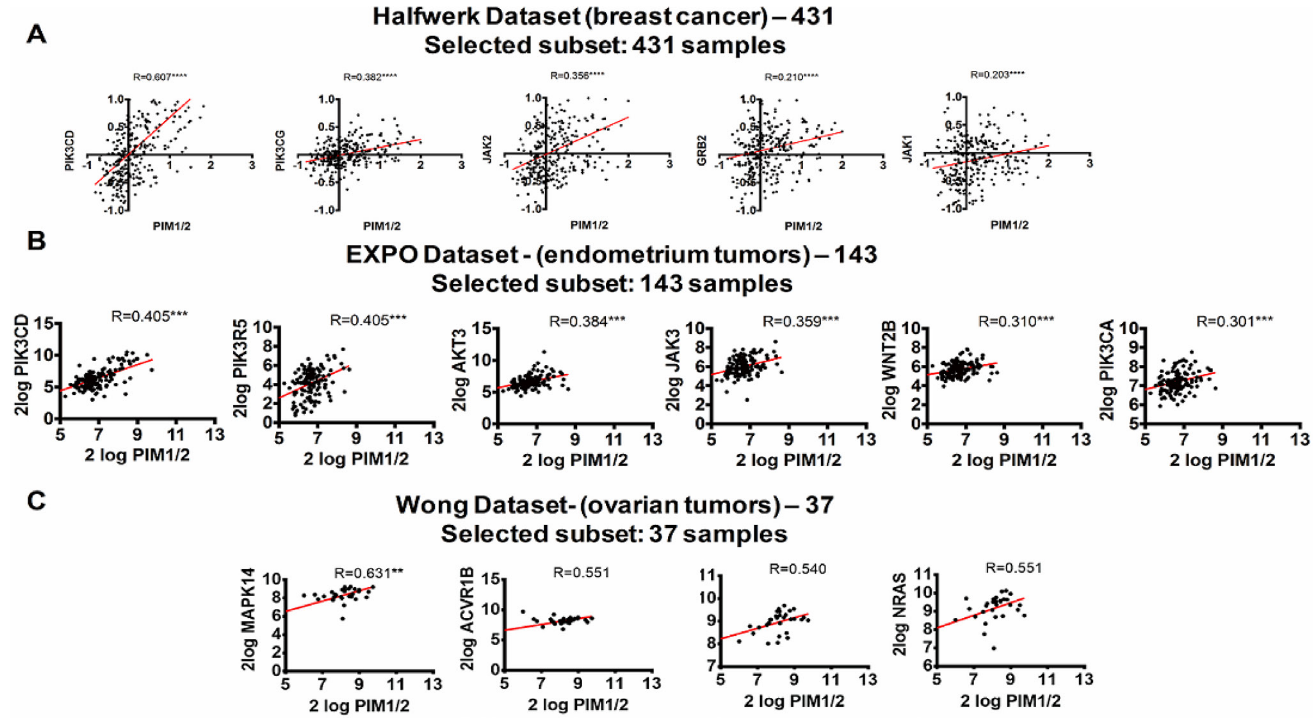

Supplementary Figure 4: One to one Pearson's correlation of the list of stem-dependent genes signature described in the heatmaps displayed in figure 6 of human breast, endometrium and ovary tumors databases analysed.

Supplementary Table 1: Common genes correlating with PIM1 and PIM2 expression in breast, ovarian and endometrium tumors. See Supplementary_Table_1

Supplementary Table 2: GO enrichment for process of genes correlating with PIM1 and PIM2 expression in all three breast, ovarian and endometrium tumors

Goid	Term	Corrected_*P* value
**GO:0009617**	**response to bacterium**	3,43E+07
**GO:0002376**	**immune system process**	7,67E+07
**GO:0043207**	**response to external biotic stimulus**	2,71E+08
**GO:0051707**	**response to other organism**	2,71E+08
**GO:0009607**	**response to biotic stimulus**	4,28E+08
**GO:0032496**	**response to lipopolysaccharide**	4,33E+07
**GO:0006952**	**defense response**	4,68E+08
**GO:0006955**	**immune response**	5,91E+07
**GO:0002237**	**response to molecule of bacterial origin**	6,40E+06
**GO:0034097**	**response to cytokine**	3,32E+09
**GO:0048583**	**regulation of response to stimulus**	5,15E+09
**GO:0009605**	**response to external stimulus**	0.000132246974172086
**GO:0007165**	**signal transduction**	0.000185186238256669
**GO:0051716**	**cellular response to stimulus**	0.000192915225072843
**GO:0007166**	**cell surface receptor signaling pathway**	0.000194146704417251
**GO:0071345**	**cellular response to cytokine stimulus**	0.000200820221676121
**GO:0050896**	**response to stimulus**	0.000219101934006273
**GO:0033622**	**integrin activation**	0.000229550239127017
**GO:0045087**	**innate immune response**	0.000371687962152313
**GO:0002682**	**regulation of immune system process**	0.000400231755999877
**GO:0044700**	**single organism signaling**	0.000571677399544299
**GO:0048584**	**positive regulation of response to stimulus**	0.000606581231880176
**GO:0023052**	**signaling**	0.000607020301368781
**GO:0007154**	**cell communication**	0.000615016495071497
**GO:0002684**	**positive regulation of immune system process**	0.00153489328532374
**GO:1901700**	**response to oxygen-containing compound**	0.00195704193579501
**GO:0051704**	**multi-organism process**	0.00253810803158615
**GO:0048518**	**positive regulation of biological process**	0.00267249757157674
**GO:0010033**	**response to organic substance**	0.00367383310482357
**GO:0006950**	**response to stress**	0.00398041612316704
**GO:1901701**	**cellular response to oxygen-containing compound**	0.00657112985458861
**GO:0033993**	**response to lipid**	0.00711211266110781
**GO:0050900**	**leukocyte migration**	0.00832471826976121
**GO:0050789**	**regulation of biological process**	0.0090034003189567
**GO:0080134**	**regulation of response to stress**	0.00925245974559772

Supplementary Table 3: Proteins of antigen processing and presentation (KEGG) (HLA and inflammation) correlating with PIM1 or PIM2 levels in breast cancer

	Halfwerk dataset	
	r-value	***P-Value***
**HLA-DOB**	0.638	< 0.01
**CD8A**	0.637	< 0.01
**CD74**	0.548	< 0.01
**HLA-DMB**	0.547	< 0.01
**HLA-DMA**	0.537	< 0.01
**HLA-DPA1**	0.517	< 0.01
**HLA-DRA**	0.517	< 0.01
**HLA-E**	0.514	< 0.01
**DPB1**	0.513	< 0.01
**CD8B**	0.505	< 0.01
**HLA-F**	0.485	< 0.01
**CTSS**	0.473	< 0.01
**IFI30**	0.469	< 0.01
**TAP1**	0.463	< 0.01
**HLA-A**	0.455	< 0.01
**KLRD1**	0.444	< 0.01
**HLA-G**	0.435	< 0.01
**HLA-B**	0.426	< 0.01
**HLA-C**	0.416	< 0.01

Supplementary Table 4: Proteins of antigen processing and presentation (KEGG) (HLA and inflammation) correlating with PIM1 or PIM2 levels in endometrial cancer

	GSE2109	
	r-value	***P-Value***
**CD4**	0.205	< 0.01
**CTSS**	0.280	< 0.01
**CD74**	0.193	< 0.01
**HLA-DMA**	0.193	< 0.01
**HLA-DRA**	0.217	< 0.01
**HLA-DPB1**	0.217	< 0.01
**HLA-DOA**	0.195	< 0.01
**TAP2**	0.232	< 0.01
**TAP1**	0.237	< 0.01
**HLA-F**	0.217	< 0.01
**CTSB**	0.235	< 0.01
**CD8A**	0.109	0.09
**IFNG**	0.166	0.02
**PSME3**	0.042	0.55

Supplementary Table 5: Proteins of antigen processing and presentation (KEGG) (HLA and inflammation) correlating with PIM1 or PIM2 levels in ovarian cancer

	GSE40595	
	r-value	***P-Value***
**PDIA2**	0.664	< 0.01
**CTSB**	0.591	0.01
**HLA-DOA**	0.578	0.01
**B2M**	0.573	< 0.01
**HLA-C**	0.566	< 0.01
**CTSL**	0.564	< 0.01
**HLA-G**	0.533	0.02
**HLA-DPA1**	0.510	0.02
**HLA-F**	0.485	0.03
**HLA-A**	0.485	0.03
**TAP2**	0.483	0.03
**HSP90AA1**	0.477	0.03
**HLA-B**	0.458	0.04
**HSPA5**	0.438	0.05

Supplementary Table 6: Proteins of proteins of pathways controlling stemness and pluripotency (KEGG) correlating with PIM1 or PIM2 levels in breast cancer

	Halfwerk dataset	
	r-value	***P-Value***
**PIK3CD**	0.607	< 0.01
**PIK3CG**	0.382	< 0.01
**JAK2**	0.356	< 0.01
**GRB2**	0.210	< 0.01
**JAK1**	0.203	< 0.01

Supplementary Table 7: Proteins of pathways controlling stemness and pluripotency (KEGG) endometrial cancer

	GSE2109	
	r-value	***P-Value***
**PIK3CD**	0.405	< 0.01
**PIK3R5**	0.405	< 0.01
**AKT3**	0.384	< 0.01
**JAK3**	0.359	< 0.01
**WNT2B**	0.310	< 0.01
**PIK3CA**	0.301	< 0.01

Supplementary Table 8: Proteins of Pathways controlling stemness and pluripotency (KEGG) correlating with PIM1 or PIM2 levels in Ovarian Cancer

	GSE40595	
	r-value	***P-Value***
**MAPK14**	0.631	0.02
**ACVR1B**	0.553	0.07
**GRB2**	0.540	0.06
**NRAS**	0.506	0.08
