# Supplementary material for: Inflammation and stem markers association to PIM1/PIM2 kinase-induced tumors in breast and uterus
Source: Oncotarget. 2017 Jul 22;8(35):58872–86. doi: 10.18632/oncotarget.19438 (PMC5601700; doi:10.18632/oncotarget.19438)
Supplement: Supplementary file 2 [file oncotarget-08-58872-s002.docx]

**Supplementary Table 1:** Common genes correlating with PIM1 and PIM2 expression in Breast, Ovarian and Endometrium tumors

| **Common 3 tumor types** | **Common Breast and Endometrium** | **Common Ovarian and Breast** | **Common ovarian and endometrium** |
| --- | --- | --- | --- |
| 80 | 42 | 286 | 119 |
| ITGA5 | LHFPL2 | FSTL1 | MYO9B |
| GFPT2 | PTPN22 | SAMD4A | C5AR1 |
| CD38 | SELL | NCF4 | SLCO2B1 |
| MZB1 | CCL19 | SERPINF1 | COL1A1 |
| PTRF | FCN1 | CST7 | DUSP5 |
| CD53 | SH2D2A | SAMSN1 | PXDN |
| HK3 | ADCY3 | PLCL2 | TNFAIP6 |
| TFEC | IGLJ3 | EMILIN1 | NCF1 |
| SLA | FAM49A | CD6 | CD93 |
| CPVL | IGLL5 | CHST11 | BGN |
| TNFRSF17 | CCL18 | HLA-J | MAGI2-AS3 |
| ARHGAP25 | IGKC | QKI | ADAMTS12 |
| CLIP2 | FXYD5 | MAN2B1 | FCGR2A |
| GCH1 | SLAMF7 | NFIL3 | AXL |
| SOCS3 | CXCL10 | GLG1 | TCEAL2 |
| GIMAP4 | SLC1A3 | UBE2L6 | PAPD7 |
| GUSBP11 | IL2RG | ARHGAP15 | LINC01094 |
| SLC7A7 | CD40 | BTK | WISP1 |
| IL10RA | CD27 | MICALL1 | FCRL5 |
| MARCO | WAS | KIAA0226L | EHD1 |
| TLE4 | PTPN7 | APBA2 | CLEC5A |
| C1QA | CD79A | SIRPG | PDGFRA |
| IFNAR2 | CD48 | CASP1 | KLHL5 |
| ITGAL | DENND1C | UBE2J1 | LINC00312 |
| RBMS1 | AIM2 | ELF4 | TREM1 |
| COL6A2 | LAIR1 | NT5E | FAM20C |
| SPHK1 | IGHD | NCK2 | BNC2 |
| SLAMF8 | CD2 | SIGLEC1 | AKT3 |
| SRGN | PLEKHO2 | FLI1 | TYMP |
| RAP2B | NCKAP1L | MCM5 | FAM65A |
| IRF4 | GZMB | IL15RA | JAM3 |
| RGS2 | LCP2 | FOLR2 | SLC16A7 |
| PDPN | RELB | GIMAP6 | DOCK8 |
| CD163 | CCL13 | ICAM2 | SNX10 |
| CCR1 | ADAMDEC1 | TNFAIP3 | KLF6 |
| TUBB6 | LILRB4 | FOXO1 | GPR176 |
| IGLC1 | SIT1 | CRLF3 | GREM1 |
| TNFRSF1B | SASH3 | IL18RAP | ITGAM |
| IL7R | CXCL9 | GNLY | PECAM1 |
| TIMP2 | INPP5D | STK10 | LATS2 |
| HSD11B1 | IL16 | MRAS | FAM105A |
| TRBC1 | CYAT1 | HMHA1 | NUDT11 |
| DDR2 |  | MFGE8 | RIN3 |
| CRISPLD2 |  | SAMHD1 | AEBP1 |
| HLX |  | EFHD2 | SLC25A19 |
| PLEKHO1 |  | NDRG1 | INSR |
| EVI2A |  | DZIP1 | LOC101928173 |
| SLAMF1 |  | LAT | COL5A1 |
| IGLV1-44 |  | FBLN2 | PPP1R18 |
| HCK |  | RASGRP3 | C10orf54 |
| DSE |  | KIAA0922 | CSF1R |
| CCL4 |  | CHST12 | ITPRIP |
| PIK3CD |  | SLC15A3 | HCST |
| MRC1 |  | RHOG | GLT8D2 |
| PTGER4 |  | HERC5 | FAM63B |
| IFFO1 |  | ICAM3 | SERPINB9 |
| CNN2 |  | BICD2 | RASSF5 |
| SIRPA |  | TRAF3IP3 | STON1 |
| SELPLG |  | CD8B | FN1 |
| EMP3 |  | RARRES1 | CSTA |
| VAMP5 |  | FAT1 | NUAK1 |
| CYTIP |  | FAM46C | THY1 |
| ROBO3 |  | CXCR6 | FAM78A |
| PLEK |  | IGSF6 | POTEKP |
| RFTN1 |  | LY9 | SLC31A2 |
| CLEC2B |  | SFT2D2 | EOMES |
| IGLL3P |  | AMPD2 | COLEC12 |
| IGHM |  | HLA-C | PLXND1 |
| CCR2 |  | LBH | PLPP4 |
| CD3D |  | FKBP11 | SASH1 |
| PXDC1 |  | MAP7D1 | NAMPT |
| BICC1 |  | MAFF | ENOX1 |
| VCAM1 |  | LY96 | AKAP12 |
| LAPTM5 |  | CH25H | HSPA12B |
| CCR5 |  | LBR | LINC00307 |
| IRF8 |  | PTPRCAP | NID2 |
| FLNA |  | NMB | LHFP |
| VAV1 |  | MDFIC | GLS |
| CSF2RB |  | IRF1 | LOX |
| HCLS1 |  | MSN | ZNF469 |
|  |  | SH2D1A | CFLAR |
|  |  | DUSP22 | COLGALT1 |
|  |  | GPNMB | C11orf96 |
|  |  | RGS16 | TSPAN4 |
|  |  | MSL3 | PLAU |
|  |  | NFATC1 | DPYSL2 |
|  |  | RAC2 | RAB8B |
|  |  | PLTP | STX2 |
|  |  | BTN3A2 | ARHGAP30 |
|  |  | HBEGF | LAIR2 |
|  |  | MGAT1 | SLC2A3 |
|  |  | CRYAB | RGS19 |
|  |  | DUSP2 | GM2A |
|  |  | NCF2 | MYO5A |
|  |  | TAP1 | VSIG4 |
|  |  | ARHGAP4 | RND3 |
|  |  | ARL4C | SNX20 |
|  |  | FCGR1B | RAB31 |
|  |  | PLAGL1 | PBX3 |
|  |  | BATF3 | FERMT2 |
|  |  | MS4A6A | CPNE8 |
|  |  | TRPV2 | DENND2C |
|  |  | EGR2 | IGK |
|  |  | VIM | JUNB |
|  |  | HLA-DMB | METRNL |
|  |  | HLA-A | ICAM1 |
|  |  | PPP2R5C | RECK |
|  |  | GBP2 | THBS2 |
|  |  | NINJ2 | CTSL |
|  |  | ITM2C | ETS1 |
|  |  | FCHSD2 | SIGLEC10 |
|  |  | SUN2 | MS4A7 |
|  |  | AIF1 | SERPINE1 |
|  |  | IL6 | ANGPTL2 |
|  |  | STAG3 | SYNPO |
|  |  | UBASH3A | CEBPB |
|  |  | ACAP1 | JCHAIN |
|  |  | LSP1 | SPOCD1 |
|  |  | PTGDS | MMP9 |
|  |  | LYN |  |
|  |  | SERPINE2 |  |
|  |  | LGMN |  |
|  |  | GPR183 |  |
|  |  | HIVEP2 |  |
|  |  | LILRB1 |  |
|  |  | PIK3CG |  |
|  |  | TBC1D1 |  |
|  |  | CCL8 |  |
|  |  | CORO1A |  |
|  |  | USB1 |  |
|  |  | BTN3A3 |  |
|  |  | C1QB |  |
|  |  | TRAC |  |
|  |  | RHBDF2 |  |
|  |  | S100A10 |  |
|  |  | WWTR1 |  |
|  |  | NFKBIE |  |
|  |  | MYO1F |  |
|  |  | HLA-B |  |
|  |  | CSRP2 |  |
|  |  | PPM1F |  |
|  |  | TCF7 |  |
|  |  | TDO2 |  |
|  |  | CTSH |  |
|  |  | MAP4K4 |  |
|  |  | EOGT |  |
|  |  | STK38 |  |
|  |  | AOAH |  |
|  |  | NOTCH1 |  |
|  |  | DAPK1 |  |
|  |  | ARHGAP22 |  |
|  |  | CRTAM |  |
|  |  | LILRB2 |  |
|  |  | FXYD6 |  |
|  |  | TLR1 |  |
|  |  | NPC2 |  |
|  |  | C3 |  |
|  |  | BTN3A1 |  |
|  |  | FABP5 |  |
|  |  | YME1L1 |  |
|  |  | SERPING1 |  |
|  |  | NFKB2 |  |
|  |  | ADAM19 |  |
|  |  | FYB |  |
|  |  | LDHB |  |
|  |  | GMFG |  |
|  |  | RGL1 |  |
|  |  | LIF |  |
|  |  | IL27RA |  |
|  |  | TMEM140 |  |
|  |  | IFI16 |  |
|  |  | RASSF4 |  |
|  |  | FYN |  |
|  |  | SGK1 |  |
|  |  | ETV7 |  |
|  |  | IGFLR1 |  |
|  |  | PTGS2 |  |
|  |  | IL21R |  |
|  |  | CD200 |  |
|  |  | LST1 |  |
|  |  | CD79B |  |
|  |  | ACOT9 |  |
|  |  | PLS3 |  |
|  |  | CD86 |  |
|  |  | NNMT |  |
|  |  | STAT1 |  |
|  |  | SSR4 |  |
|  |  | BCL2A1 |  |
|  |  | PRDM1 |  |
|  |  | BIN1 |  |
|  |  | PTPRC |  |
|  |  | MYO1E |  |
|  |  | CD14 |  |
|  |  | C1S |  |
|  |  | TCF7L1 |  |
|  |  | CECR1 |  |
|  |  | EBI3 |  |
|  |  | NXN |  |
|  |  | PARP12 |  |
|  |  | BTN2A2 |  |
|  |  | PIP4K2A |  |
|  |  | CXCR3 |  |
|  |  | FSCN1 |  |
|  |  | CLEC7A |  |
|  |  | GBP1 |  |
|  |  | HLA-DPB1 |  |
|  |  | CD74 |  |
|  |  | EVI2B |  |
|  |  | AKR1B1 |  |
|  |  | HLA-G |  |
|  |  | SOCS1 |  |
|  |  | CELF2 |  |
|  |  | IL4R |  |
|  |  | CCL5 |  |
|  |  | SH3BGRL3 |  |
|  |  | LYZ |  |
|  |  | ADM |  |
|  |  | LAP3 |  |
|  |  | ICOS |  |
|  |  | WIPF1 |  |
|  |  | TPST1 |  |
|  |  | LPIN1 |  |
|  |  | IL18R1 |  |
|  |  | CLEC10A |  |
|  |  | TCN2 |  |
|  |  | PLAUR |  |
|  |  | SIK1 |  |
|  |  | TMEM176B |  |
|  |  | C1QTNF1 |  |
|  |  | MLXIP |  |
|  |  | PLOD1 |  |
|  |  | FGL2 |  |
|  |  | ANXA1 |  |
|  |  | C1R |  |
|  |  | PRKCDBP |  |
|  |  | DOCK2 |  |
|  |  | TRIM22 |  |
|  |  | GPX7 |  |
|  |  | ATF3 |  |
|  |  | IKZF1 |  |
|  |  | HLA-E |  |
|  |  | FLT3LG |  |
|  |  | RAI14 |  |
|  |  | CFH |  |
|  |  | NECAP2 |  |
|  |  | EMP1 |  |
|  |  | CCDC109B |  |
|  |  | CSK |  |
|  |  | CTSS |  |
|  |  | LCK |  |
|  |  | HLA-DPA1 |  |
|  |  | WARS |  |
|  |  | LTB |  |
|  |  | MPP1 |  |
|  |  | DRAM1 |  |
|  |  | RGCC |  |
|  |  | DEF6 |  |
|  |  | CCL2 |  |
|  |  | ADA |  |
|  |  | MAST1 |  |
|  |  | MX2 |  |
|  |  | KIFC3 |  |
|  |  | ZDHHC18 |  |
|  |  | HLA-F |  |
|  |  | MOXD1 |  |
|  |  | COTL1 |  |
|  |  | SERPINH1 |  |
|  |  | HERPUD1 |  |
|  |  | SLC36A1 |  |
|  |  | LRRK1 |  |
|  |  | RARRES2 |  |
|  |  | GZMA |  |
|  |  | DOK3 |  |
|  |  | RASSF2 |  |
|  |  | CD52 |  |
|  |  | LGALS9 |  |
|  |  | ZEB2 |  |
|  |  | APOBEC3C |  |
|  |  | UPP1 |  |
|  |  | CLIC2 |  |
|  |  | CD69 |  |
|  |  | PSMB10 |  |
|  |  | PRKCA |  |
|  |  | RPS6KA3 |  |
|  |  | PSMB9 |  |
|  |  | CTSC |  |
